# Effects of Carbohydrate and Dietary Fiber Intake, Glycemic Index and Glycemic Load on HDL Metabolism in Asian Populations

**DOI:** 10.14740/jocmr1884w

**Published:** 2014-07-28

**Authors:** Hidekatsu Yanai, Hisayuki Katsuyama, Hidetaka Hamasaki, Shinichi Abe, Norio Tada, Akahito Sako

**Affiliations:** a Department of Internal Medicine, National Center for Global Health and Medicine Kohnodai Hospital, Chiba, Japan; b The Jikei University School of Medicine, Tokyo, Japan

**Keywords:** Carbohydrate, Dietary fiber, Glycemic index, Glycemic load, High-density lipoprotein

## Abstract

High-density lipoprotein (HDL) is a lipoprotein which has anti-atherogenic property by reverse cholesterol transport from the peripheral tissues to liver. Low HDL-cholesterol (HDL-C) levels are associated with the development of coronary artery diseases (CADs). Various epidemiological studies have suggested that the development of CAD increase in individuals with less than 40 mg/dL of HDL-C. In spite of accumulation of evidences which suggest a significant association between low HDL-C and cardiovascular diseases, effects of dietary factors on HDL metabolism remained largely unknown. There may be interracial differences in effects of dietary factors on HDL metabolism. Here we reviewed published articles about effects of carbohydrate and dietary fiber intake, glycemic index (GI) and glycemic load (GL), on HDL-C metabolism, regarding meta-analyses and clinical studies performed in Asian population as important articles. Low carbohydrate intake, GI and GL may be beneficially associated with HDL metabolism. Dietary fiber intake may be favorably associated with HDL metabolism in Asian populations.

## Introduction

High-density lipoprotein (HDL) plays a role in reverse cholesterol transport from the peripheral tissues to liver, suppressing cholesterol accumulation in the peripheral tissue. Since HDL is a lipoprotein which has anti-atherogenic property, low HDL-cholesterol (HDL-C) level induces the development of coronary artery diseases (CADs), and high HDL-C level is associated with reduction of incidence of CAD [1, 2]. Various epidemiological studies have suggested that the development of CAD increase in individuals with less than 40 mg/dL of HDL-C [1, 3-5]. In a large scale cohort study of the relationship between serum cholesterol concentration and coronary events with low-dose simvastatin therapy in Japanese patients with hypercholesterolemia, there was higher mortality in patients with an HDL-C concentration < 40 mg/dL compared with those whose average HDL-C concentration was between 40 and 49 mg/dL by 30% [6]. According to these data, low HDL-C was defined as HDL-C concentration < 40 mg/dL in Japan. Low HDL-C is also associated with cerebrovascular diseases (CVDs), and HDL-C levels were inversely correlated with the development of death due to CVD [7].

In spite of accumulation of the data suggesting a significant influence of low HDL-C on CAD and CVD, effects of dietary factors on HDL-C remained unknown. We should also consider the existence of interracial differences in effects of dietary factors on HDL metabolism. Here we reviewed published articles about effect of dietary factors, especially, carbohydrate and dietary fiber intake, glycemic index (GI) and glycemic load (GL), on HDL metabolism. We regarded systematic review and meta-analysis and also clinical studies which were performed in Asian populations as important articles in this review.

## Effects of Carbohydrate Intake on HDL-C

The meta-analysis demonstrated that the low-carbohydrate diet (energy from carbohydrate ≤ 45% of total energy) increased HDL-C by 3.3 mg/dL as compared with the low fat diet (energy from fat ≤ 30% of total energy) [8] (Table 1). In the meta-analysis investigating the association between the intake of diet including milk fat and coronary risk factors, the substituting saturated fat from whole milk and butter for carbohydrate or unsaturated fatty acids increased HDL-C [9]. Hession et al performed the meta-analysis for comparison between low-carbohydrate diets and low-fat/low-calorie diets [10]. In this study, adult populations with mean or median body mass index (BMI) ≥ 28 kg/m^2^ were included as participants studied. There were significant differences between the groups for HDL-C, favoring the low-carbohydrate diet. In the systematic review by Crawford et al, the low-carbohydrate diets raised HDL-C by approximately 10% [11]. The meta-analysis was also performed to elucidate the effect of replacing dietary fat with carbohydrate on glucose and lipid metabolism in patients with type 2 diabetes. This meta-analysis including 19 randomized trials (n = 306) investigated the effects of a low-fat, high-carbohydrate (LFHC) diet and a high-fat, low-carbohydrate (HFLC) diet [12]. Median diet compositions of carbohydrate/fat in the LFHC and HFLC diets were 58%/24% and 40%/40%, respectively. The LFHC diet significantly lowered HDL-C by 6% (P < 0.001) compared with the HFLC diet. Nordmann et al used the Cochrane Collaboration search strategy to identify trials comparing the effects of low-carbohydrate diets without restriction of energy intake vs. low-fat diets in individuals with a BMI of at least 25 kg/m^2^ [13]. Five trials including a total of 447 individuals fulfilled the study inclusion criteria. After 6 months, HDL-C changed more favorably in individuals assigned to the low-carbohydrate diets as compared with the low-fat diets. Weighted mean difference for HDL-C was 4.6 mg/dL (95% CI; 1.5 - 8.1 mg/dL). Mensink et al calculated the effect of changes in carbohydrate and fatty acid intake on serum lipids, reviewing 27 controlled trials published between 1970 and 1991 [14]. They found that all fatty acids elevated HDL-C when substituted for carbohydrates.

**Table 1:** Meta-Analysis and Systematic Reviews About Effects of Carbohydrate Intake on HDL-C

Authors	Study design	Subjects studied	Results/conclusions
Hu et al [8]	Low-carbohydrate diets (≤ 45%) vs. low-fat diets (≤ 30%) on metabolic risk factors	2,788 participants	Compared with low-fat diets, low-carbohydrate diets induced a greater increase in HDL-C (3.3 mg/dL; 95% CI, 1.9 - 4.7)
Huth et al [9]	The relationship between milk fat containing dairy foods and cardiovascular risk factors		A diet higher in saturated fat from whole milk and butter increase HDL-C when substituted for carbohydrates or unsaturated fatty acids
Hession et al [10]	Low-carbohydrate diets vs. low-fat/low-calorie diets	Adults with BMI ≥ 28 kg/m^2^	There were significant differences between the groups for HDL-C, favoring the low-carbohydrate diet
Crawford et al [11]	Systemic review on dietary factors on metabolic parameters		Low-carbohydrate diets raise HDL-C by approximately 10%
Kodama et al [12]	Influence of fat and carbohydrate proportions on metabolic parameters	306 patients with type 2 diabetes	LFHC diet significantly lowered HDL-C by 6% (P < 0.001) compared with HFLC diet
Nordmann et al [13]	Low-carbohydrate vs. low-fat diets on cardiovascular risk factors	447 individuals	HDL-C changed more favorably in individuals assigned to low-carbohydrate diets after 6 months by 4.6 mg/dL (95% CI, 1.5 - 8.1)
Mensink et al [14]	Effect of changes in carbohydrate and fatty acid intake on serum lipid and lipoprotein levels	27 controlled trials	All fatty acids elevated HDL-C when substituted for carbohydrates

BMI: body mass index; HDL-C: high-density lipoprotein-cholesterol; HFLC: high-fat low-carbohydrate; LFHC: low-fat high-carbohydrate.

In spite of accumulation of such as meta-analysis, evidence obtained from clinical trials performed in Asian populations is very limited. We found the clinical study performed by Miyashita et al in Japan [15]. They investigated the effects of low-carbohydrate diet on glucose and lipid metabolism in obese subjects with type 2 diabetes. Subjects studied were randomly assigned to take a low-calorie and low-carbohydrate diet (n = 11, 1,000 kcal per day, protein:carbohydrate:fat = 25:40:35) or a low-calorie and high-carbohydrate diet (n = 11, 1,000 kcal per day, protein:carbohydrate:fat = 25:65:10) for 4 weeks. HDL-C increased in the low-carbohydrate diet group but not in the high-carbohydrate diet group (+15% vs. 0%, P < 0.01).

Evidences obtained from meta-analyses/systematic reviews and clinical trials in Asian populations strongly suggest that low-carbohydrate intake is beneficially associated with HDL-C metabolism.

## Effects of Dietary Fiber Intake on HDL-C

Talati et al performed a systematic literature search to determine the association between consumption of barley and changes in plasma lipids in healthy and hypercholesterolemic men and women [16]. They found eight trials (n = 391) of 4 - 12 weeks’ duration evaluating the lipid-reducing effects of barley. The use of barley did not appear to significantly alter HDL-C (P = 0.07), concluding that barley-derived beta-glucan appears not to affect HDL metabolism. Anderson et al reviewed international nutrition recommendations with a special emphasis on carbohydrate and dietary fiber in individuals with diabetes mellitus [17]. For diabetic subjects, the moderate carbohydrate and high-fiber diets compared to the moderate carbohydrate and low-fiber diets are associated with significantly lower values for HDL-C. The high-carbohydrate and high-fiber diets compared to the moderate-carbohydrate and low-fiber diets are associated with lower values for HDL-C.

Several clinical trials to study effects of dietary fiber on HDL-C were performed in Asian populations (Table 2). Zhang et al investigated the impact of oat consumption on cholesterol levels in Chinese adults with mild to moderate hypercholesterolemia [18]. The oat group consumed 100 g of instant oat cereal versus the control group who consumed 100 g of wheat flour-based noodles daily for 6 weeks. Dietary fiber intake increased significantly in the oat group compared to the control group at the end of the 6-week intervention. HDL-C decreased significantly in the control group versus the oat group. Singh et al performed a randomized, single-blind, controlled trial to examine the effects of guava fruit intake in patients with essential hypertension [19]. Seventy-two patients were assigned to take a soluble fiber and a potassium-rich diet containing daily 0.5 - 1.0 kg of guava (group A) and 73 patients to their usual diet (group B). After 4 weeks of follow-up, increased intake of soluble dietary fiber was associated with an insignificant increase in HDL-C (4.6%). They performed another study. Sixty-one and 59 patients with essential hypertension were administered guava fruit preferably before meals in a foods-to-eat (group A) approach rather than foods-to-restrict (group B), respectively, in a randomized and single-blind fashion for 12 weeks [20]. There was a significant net increase in HDL-C (8.0%) after 12 weeks of guava fruit ingestion. In the study by Zhang et al, a total of 110 elderly people with hyperlipidemia were randomly assigned to the experimental group who consumed an ordinary diet plus foods containing refined konjac meal, and the control group who consumed only the ordinary diet for 45 days [21]. At the end of the trial, HDL-C significantly elevated (P < 0.01) in the experimental group.

**Table 2:** Clinical Trials to Study Effects of Dietary Fiber Intake on HDL-C, Performed in Asian Populations

Authors	Nationality of subjects	Study design	Subjects studied	Results/conclusions
Zhang et al [18]	China	Daily 100 g of instant oat cereal vs. 100 g of wheat flour-based noodles for 6 weeks	Adults with mild to moderate hypercholesterolemia	HDL-C decreased significantly in the control group vs. the oat group
Singh et al [19]	India	Soluble dietary fiber and a potassium-rich diet containing 0.5 - 1.0 kg of guava daily (group A) vs. an usual diet (group B) for 4 weeks	145 hypertensive patients	An insignificant increase in HDL-C (4.6%) with a mild increase in TC/ HDL-C in group A patients compared with group B
Singh et al [20]	India	Guava fruit preferably before meals in a foods-to-eat approach rather than foods-to-restrict, in a randomized and single-blind fashion for 12 weeks	120 patients with essential hypertension	A significant net increase in HDL-C (8.0%) after 12 weeks of guava fruit substitution
Zhang et al [21]	China	An ordinary diet plus foods containing refined konjac meal vs. the ordinary diet for 45 days	110 elderly people with hyperlipidemia	In the experimental group, HDL-C was significantly elevated (P < 0.01). In the control group, the change in HDL-C was insignificant. The difference in HDL-C between the two groups was statistically significant

HDL-C: high-density lipoprotein-cholesterol; TC: total cholesterol.

According to results by meta-analyses, we could not determine whether dietary fiber intake is beneficially associated with HDL-C metabolism, or not. Evidences obtained from clinical trials performed in Asian populations suggest that dietary fiber intake may be beneficially associated with HDL-C metabolism. However, we should perform further studies to understand whether dietary fiber intake is correlated with elevation of HDL-C independently of other nutrients such as carbohydrate and vitamins.

## Effects of GI and GL on HDL-C

Recently, the role of GI and GL in the healthy state, in pre-diabetic and diabetic state has been discussed [22]. Carbohydrate-rich foods are classified on the basis of their effects on postprandial glucose excursion, as indicated by their GI, which is calculated by dividing the incremental area under the curve of blood glucose concentrations measured after the ingestion of a portion of a test food containing 50 g carbohydrate by the incremental blood glucose area achieved with a portion of a reference food, and expressed as a percentage [23]. Although dietary fiber is not always associated with low GI, dietary fiber-rich foods generally have a low GI [24, 25]. Postprandial blood glucose levels are influenced not only by GI, but also the amount of carbohydrate intake. Therefore, the GL which is calculated by the GI (amount of carbohydrate contained in food) has been developed to better represent both the quantity and the quality of the carbohydrate intake [26].

Goff et al conducted the meta-analysis of randomized controlled trials of low-GI diets on blood lipids, by using OVID Medline, Embase and Cochrane library [27]. Random effects meta-analyses were performed on 28 trials comparing low- with high-GI diets over at least 4 weeks (n = 1,272). There were no effects of low-GI diets on HDL-C.

Three cross-sectional studies to investigate effects of GI and GL on HDL-C were performed in Asian populations (Table 3). Choi et al studied the association between dietary carbohydrate and low HDL-C prevalence in Korean adults (n = 9,947), using the data from the Fourth Korea National Health and Nutrition Examination Survey [28]. Total carbohydrate intake (g/day), percentage of energy from carbohydrate, GI and GL were divided into quintiles. Odds ratios for having low HDL-C in the highest quintile were 1.66 (95% CI, 1.24 - 2.22) for total carbohydrate, 1.34 (1.02 - 1.75) for percentage of energy from carbohydrate, and 1.54 (1.17 - 2.03) for GL in men as compared with the second quintile as a reference. Odds ratio for low HDL-C was 1.38 (1.12 - 1.71) for percentage of energy from carbohydrate in women. Low HDL-C is associated with high-carbohydrate intake without regard to energy or fat intake in Korean population. Murakami et al examined the cross-sectional associations between dietary GI and GL and metabolic risk factors in healthy Japanese women (n = 1,354) [29]. Dietary GL was independently negatively correlated with HDL-C. Amano et al studied the correlation between dietary GI, GL and CVD risk factors in 32 Japanese women aged 52.5 ± 7.2 years old [30]. In the lowest GI tertile, the highest concentration of HDL-C was observed (P < 0.01). In the lowest GL tertile, the highest concentration of HDL-C was observed (P < 0.05).

**Table 3:** Clinical Trials to Study Effects of GI and GL on HDL-C, Performed in Asian Populations

Authors	Nationality of subjects	Study design	Subjects studied	Results/conclusions
Choi et al [28]	Korea	The association between dietary carbohydrates and low HDL-C prevalence	A total of 9,947 Korean adults older than 20 years	Odds ratios for having low HDL-C in the highest quintile were 1.66 (95% CI, 1.24 - 2.22) for total carbohydrate, 1.34 (1.02 - 1.75) for percentage of energy from carbohydrate, and 1.54 (1.17 - 2.03) for GL in men as compared with the second quintile as a reference. Odds ratio for low HDL-C was 1.38 (1.12 - 1.71) for percentage of energy from carbohydrate in women.
Murakami et al [29]	Japan	The associations between dietary GI and GL and metabolic risk factors	1,354 Japanese female farmers aged 20 - 78 years from five regions of Japan	Dietary GL was independently negatively correlated with HDL-C (n = 1,354; P = 0.004)
Amano et al [30]	Japan	The associations between dietary GI, GL and CVD risk factors	A total of 32 women aged 52.5 ± 7.2 years participated in the weight-reduction program	In the lowest GI tertile, the highest concentration of HDL-C was observed (P < 0.01). In the lowest GL tertile, the highest concentration of HDL-C was observed (P < 0.05)

CI: confidence interval; CVD: cardiovascular disease; GI: glycemic index; GL: glycemic load; HDL-C: high-density lipoprotein-cholesterol.

Based on the data obtained from clinical trials performed in Asian populations, low GI and low GL may be beneficially associated with HDL metabolism.
